# Incorporating costing study results into district and service planning to enhance immunization programme performance: a Zambian case study

**DOI:** 10.1093/heapol/czz039

**Published:** 2019-06-03

**Authors:** Isabelle Feldhaus, Carl Schütte, Francis D Mwansa, Masauso Undi, Stanley Banda, Chris Suharlim, Nicolas A Menzies, Logan Brenzel, Stephen C Resch, Anthony Kinghorn

**Affiliations:** 1 Department of Global Health and Population, Harvard T.H. Chan School of Public Health, Boston, MA, USA; 2 Strategic Development Consultants, Pietermaritzburg, South Africa; 3 Department of Public Health, Ministry of Health, Plot 12193, Woodlands Chalala, Lusaka, Zambia; 4 Independent consultant, 35 Nalikwanda Road, Woodlands, Lusaka, Zambia; 5 Independent consultant, Plot 34270, Shantumbu Road, Hillview Park, Lusaka, Zambia; 6 Center for Health Decision Science, Harvard T.H. Chan School of Public Health, 718 Huntington Ave, Boston, MA, USA; 7 Bill & Melinda Gates Foundation, 500 Fifth Avenue N, Seattle, WA, USA; 8 Perinatal HIV Research Unit, Chris Hani Baragwanath Academic Hospital, Chris Hani Road, Diepkloof, Soweto, South Africa

**Keywords:** Routine immunization, costing, immunization programmes, decentralization, decentralized planning and decision-making, Zambia

## Abstract

Donors, researchers and international agencies have made significant investments in collection of high-quality data on immunization costs, aiming to improve the efficiency and sustainability of services. However, improved quality and routine dissemination of costing information to local managers may not lead to enhanced programme performance. This study explored how district- and service-level managers can use costing information to enhance planning and management to increase immunization outputs and coverage. Data on the use of costing information in the planning and management of Zambia’s immunization programme was obtained through individual and group semi-structured interviews with planners and managers at national, provincial and district levels. Document review revealed the organizational context within which managers operated. Qualitative results described managers’ ability to use costing information to generate cost and efficiency indicators not provided by existing systems. These, in turn, would allow them to understand the relative cost of vaccines and other resources, increase awareness of resource use and management, benchmark against other facilities and districts, and modify strategies to improve performance. Managers indicated that costing information highlighted priorities for more efficient use of human resources, vaccines and outreach for immunization programming. Despite decentralization, there were limitations on managers’ decision-making to improve programme efficiency in practice: major resource allocation decisions were made centrally and planning tools did not focus on vaccine costs. Unreliable budgets and disbursements also undermined managers’ ability to use systems and information. Routine generation and use of immunization cost information may have limited impact on managing efficiency in many Zambian districts, but opportunities were evident for using existing capacity and systems to improve efficiency. Simpler approaches, such as improving reliability and use of routine immunization and staffing indicators, drawing on general insights from periodic costing studies, and focusing on maximizing coverage with available resources, may be more feasible in the short-term.

**Key Messages**

- Costing studies can provide valuable insights into service delivery and highlight new priorities, but decision-making to enhance programme performance may depend on factors beyond improved availability and quality of routine cost information, such as the capacity of local managers to make decisions within a complex system.
- Despite decentralization and delegation of decision-making power, there are considerable limitations on the range of decisions that managers can make to improve immunization programme efficiency in Zambia.
- Clear conceptualization of the complex systems affecting the decision space of local managers and identification of local-level opportunities to strengthen performance are important to mitigating challenges that hinder improved programme performance.
- Improving reliability and use of routine immunization and staffing indicators with available resources may be a simpler way to improve performance than intensifying routine costing activities.

## Introduction

District- and service-level managers play a critical role in the efficient and effective functioning of primary healthcare systems, which often encompass administration of national immunization programmes. Local managers may be responsible for a range of tasks across the spectrum of vaccine delivery. These can include responding to outbreaks of vaccine-preventable diseases, organizing national immunization days, communicating with health providers and the public on the benefits of vaccines, maintaining dependable procurement and supply processes, maintaining effective surveillance systems, training immunization staff, and adhering to the most recent immunization guidelines and regulations (Sabin Vaccine Institute, XXXX, XXXX). Given their central role in responding to the day-to-day operations, bottlenecks and challenges of programme implementation, local managers have the potential to enhance immunization delivery by increasing the productivity and agility of programme operations (McKinsey & Company, 2016).

Rooted in new public management theory, improving the quality of and access to monitoring and evaluation data has been a key focus in improving the performance of public sector programmes (Blackman, 2001; Stansfield *et al.*, 2006; Wickremasinghe *et al.*, 2016). Donors, researchers and international agencies have made significant investments in the collection of high-quality data on routine immunization costs with aims to understand cost trends, predict future costs, inform financing strategies and thereby improve the efficiency and sustainability of immunization services within specific countries (Ahanhanzo *et al.*, 2015; Goguadze *et al.*, 2015; Guthrie *et al.*, 2015; Janusz *et al.*, 2015; Le Gargasson *et al.*, 2015; Maceira *et al.*, 2015; Schütte *et al.*, 2015; Valdés *et al.*, 2015; Menzies *et al.*, 2017). However, dissemination of results to district- and service-level managers often does not occur. Furthermore, improved data quality and the routine dissemination of costing information to local managers may not translate to enhanced programme performance (Stansfield *et al.*, 2006; Wickremasinghe *et al.*, 2016). Decision-making to enhance performance may depend on factors beyond improved data quality and routine data dissemination, such as the capacity and authority of local managers to make decisions as agents within a complex system (Wickremasinghe *et al.*, 2016).

Complexity theory as applied to management of public systems presents an alternative approach to understanding the factors that enable more effective decision-making by managers (Blackman, 2001; Rhodes and MacKechnie, 2003; Rhodes, 2012). This approach emphasizes the health system as a complex system characterized by dynamic relationships and dependent networks rather than the simple aggregation of its static constituent parts (Blackman, 2001; McEvoy *et al.*, 2016). Complex systems theory would suggest that the ability of local immunization managers to act will depend on the environment for decision-making. ‘Environment’ could refer to their formal authority, existing organizational structures and systems, interpersonal relationships, budget constraints and the skills and values of individual system agents.

The 7-S framework, developed by Waterman *et al.* (1980), allows for a systematic and holistic description of organizational strengths and weaknesses through distinct management dimensions as well as an understanding of the complex systems involved and more informed prioritization of areas in which interventions may improve performance. It is based on the central idea that organization effectiveness stems from strengths or limitations related to any (or many) of seven dimensions and their sub-components (e.g. systems, structure, strategy, style of leadership, staff capacity and skills, shared values and other factors) (Waterman *et al.*, 1980). Analysing the health system from this perspective can highlight those factors affecting local decision-making beyond the simple dissemination of adequate data and information. This may enhance understanding of *how* these data and information should be packaged for optimal, actionable use in a specific context, and may also lead to prioritization of system interventions that are prerequisites for the ability to act on information.

The objective of this study was to explore how district- and/or service-level immunization programme managers can use costing information to enhance planning and management to increase immunization outputs and coverage. Characterizing their constraints and opportunities within the broader health system is a first step in further understanding how managers can feasibly use costing data for improved vaccine delivery. Zambia was selected as a case study, having decentralized its health sector in the early 1990s and being one of six countries that participated in the 2014 Expanded Programme on Immunization Costing (EPIC) study (Bossert *et al.*, 2003; Brenzel *et al.*, 2015).

### EPIC study in Zambia

With a population of nearly 16 million and nearly 60% living beneath the poverty line in 2017, Zambia faces major health challenges, including a severe HIV epidemic contributing to high maternal (398 deaths per 100 000 live births) and under-five mortality (75 deaths per 1000 live births) (Central Intelligence Agency, XXXX; CSO/MoH/ICF, 2014; The World Bank Group, XXXX). Following an economic downturn in 2014, the health sector has faced increasingly limited and uncertain budget allocations alongside persistent operational challenges in service delivery. Contributing factors underlying these challenges include: inadequate numbers and skills of managers and health workers; poor morale, motivation and retention; unreliable provision of essential medicines and supplies; limited autonomy in decision-making at decentralized levels; and weak monitoring and evaluation (Friedman *et al.*, 2016; Zeng *et al.*, 2018). In this context, refining the planning, budgeting and management of limited health resources is increasingly important for Zambia to maximize their impact.

The EPIC study aimed to provide updated information on routine immunization costs, cost determinants, and new vaccine introduction costs, using standardized methodologies to enhance international comparison and interpretability of results (Brenzel *et al.*, 2015; Griffiths *et al.*, 2016). Detailed methodology and findings have been documented by Schütte *et al.* (2014, 2015). The study highlighted differences in unit costs across facilities over a range of input and activity categories and suggested inefficiencies in immunization delivery in six countries, including Zambia (Geng *et al.*, 2017; Menzies *et al.*, 2017). Reasons for differences in unit costs and efficiency of services across facilities included differences in facility workloads (e.g. number of doses or patients), discrepancies between levels of staffing and workloads, extent of outreach activities, inefficient delivery models, remote locations with high transport costs, duplication of services and underutilized staff (Schütte *et al.*, 2014). Overall, study results were interpreted to suggest that sustainability, and ability to maintain and increase coverage with available resources, would require effective facility- and district-level management of the largest cost items, particularly human resources, vaccines, travel and outreach.

### Decentralization and decision-making for health in Zambia

Decentralization is relevant to a case study examining local managers’ capacity to enhance programme performance; local managers must have some power to make decisions. The range of effective choice available to local managers can be described using the concept of ‘decision space’ (Bossert, 1998). Based on the principal-agent approach used to examine managerial, political and interpersonal decision-making, the decision space framework has primarily been used to evaluate decentralization processes in health reforms whereby a central authority has expanded the mandates at local levels (Bossert, 1998; Bossert *et al.*, 2003; Seshadri *et al.*, 2016).

Previous decentralization initiatives in the health sector in Zambia were characterized by a combination of *deconcentration* to district health officials and *delegation* of authority from the Ministry of Health (MoH) to an autonomous Central Board of Health and, to a lesser degree, the District Health Boards and District Hospital Boards (Bossert *et al.*, 2003). Despite increased decision space at district and facility levels following decentralization, District Health Management Teams still had limited choice over sources of additional revenue, remained unaccountable to local government, and could not make decisions on staff salaries or local governance structures. On the other hand, districts had moderate choice over expenditures, fees, hiring and firing, contracts with private providers, local health office organization and community participation. They also had unusually wide choice over prepayment schemes, payment mechanisms and contracting of short-term personnel (Bossert and Beauvais, 2002; Bossert *et al.*, 2003).

An analysis of vaccination coverage across 144 countries found that DTP3 and measles coverage was higher in countries with a higher degree of decentralization (Khaleghian, 2004). However, evaluation of the impact of decentralization in Zambia highlighted a significant decline in immunization rates, with this decline concentrated in the poorest, most rural and lowest population districts (Bossert *et al.*, 2003). Bossert *et al.* (2003) note that, while this decline may have been the result of decentralization, other potential causes included the decline in expenditures and disruption in donor support (Bossert *et al.*, 2003).

## Materials and methods

### Sampling and eligibility criteria

Individual and group semi-structured interviews were conducted with planners and managers at national, provincial and district levels in Zambia between October and December 2015. Only individuals working in the districts selected for EPIC data collection (i.e. Chongwe, Kabwe, Kafue, Lufwanyama, Lusaka, Masaiti, Mkushi, Ndola and Serenje) were eligible for inclusion in this study (Schütte *et al.*, 2014, 2015). At the national level, targeted informants included planning and management personnel (i.e. strategic, operational and infrastructure planning; health information; revenue and allocation to health; budgeting and expenditure control) at the MoH and the Ministry of Community Development, Maternal and Child Health (MCDMCH). Targeted informants at the provincial and district offices included provincial medical officers (PMOs), district medical officers (DMOs), maternal and child health (MCH) managers, immunization managers, health planners, provincial and district accountants, clinical care specialists and cold chain technicians. All individuals engaged in these roles and responsibilities for the Expanded Programme on Immunization (EPI) at national, provincial or district levels were eligible for inclusion in this study. PMOs and/or DMOs in each study area were contacted to organize a 1-hour group meeting with their planning and management teams according to the above criteria.

In the final sample, 13 national-level key informants were interviewed by researchers individually. Three provincial-level group discussions were held; each of these groups was comprised of four informants. Nine district-level management group discussions were conducted with an average of five participants each. All targeted informants and aforementioned districts were represented and actively participated in discussions.

### Data collection

Data collection in this study was guided by a grounded theory approach in which hypotheses and the focus of data collection and tools were adapted based on findings over the course of the study. Interview guides were tailored to type of respondent, resulting in four national–level and two provincial/district–level interview guides to obtain data on the use of costing study information on the planning and management of EPI (see Supplementary Appendix). Individual interviews were conducted at the national level to explore planning, management and other systems and practices. Group interviews were then conducted at provincial and district levels to validate and elaborate on systems and practices. Interview schedules included broad introductory questions followed by specific prompts to probe into particular aspects of responses.

Interviews were introduced by a description of the study objectives, followed by presentation of EPIC study results overall and for sampled facilities in participants’ jurisdictions, and finally, discussion. Respondents were invited to ask questions throughout the interview process. Interview schedules included broad introductory questions and open-ended rather than directive questions were used to enable participants to identify priority issues without undue influence of the interviewer. The questionnaires focused on identifying core functions, strategic priorities, planning and review processes, systems and tools, cost and efficiency determinants, and challenges for performance at each level.

In addition to individual and group interviews, a 1-day workshop was held on 3 December 2015 with representation from two districts. The workshop included three main sessions aiming to: (1) disseminate the results of routine costing of EPI in Zambia, (2) identify any underlying factors causing differences in costs between facilities and (3) design strategies for more efficient resource use for immunization. The workshop ended with a final session to gather recommendations on integrating efficiency into annual planning and management of immunization, and outline follow-up actions for participants.

Interviews and group discussions were conducted by two authors. These researchers and three additional authors conducted the workshop. Data were collected by detailed notetaking during each interview and the workshop. The two authors leading interviews took and compared notes for completeness at regular intervals throughout data collection. They also discussed emerging themes and questions; developing findings were further explored in later interviews and discussions.

A review of mentioned policy and guidance documents mentioned by key informants was also conducted (see Supplementary Appendix). This document review informed researchers’ understanding of the formal environment in which managers were making decisions for EPI and helped triangulate different stakeholders’ views of systems.

### Data analysis

Descriptive synthesis of policy documents was undertaken to understand the formal environment of decision-making among local managers for EPI. Qualitative analysis of interviews was conducted to identify perceived drivers of programme costs and efficiency as well as understand the organizational context within which managers were operating. To systematically and comprehensively explore key organization features and their relationships, responses were categorized using the dimensions outlined by the Waterman *et al.* (1980) 7-S framework:

- *Systems* (formal and informal procedures of the organization)
- *Structure* (coordination of programmes and services; management and planning structures)
- *Strategy* (health priorities; service models; guidelines)
- *Style of leadership* (delegation of authority; decentralized initiative; delegation style; accountability)
- *Staff capacity and skills* (sufficient numbers with key skills available to perform functions)
- *Shared values* (commitment to service performance; efficiency; teamwork)
- *Other factors* (additional contextual features)

### Ethical clearance

This study was the dissemination phase of the EPIC study approved by the University of Zambia Biomedical Research Ethics Committee. The National Health Research Authority authorized the dissemination of costing study results to provincial- and district-level managers through the interviewing and workshop procedures outlined above. This study was approved as exempt human research by the Institutional Review Board at the authors’ institute.

## Results

### Decision-making environment for the expanded programme on immunization in Zambia

**Figure 1:**
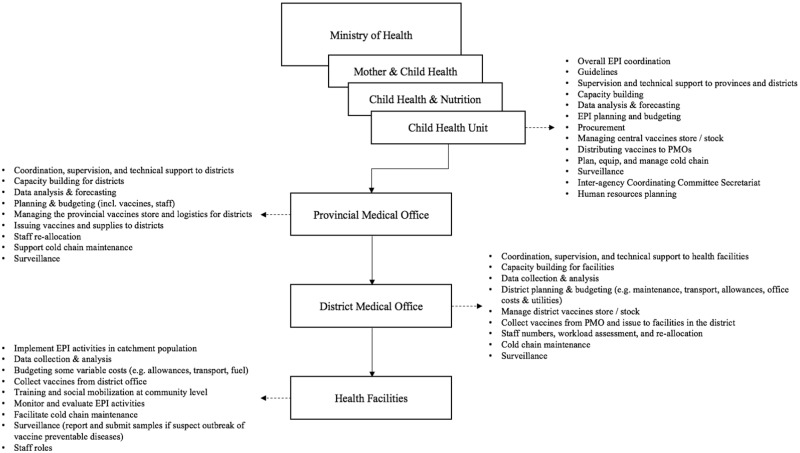
Structural organization and institutional functions of EPI in Zambia.

The review of policy and planning documents identified several key influences on the immunization programme environment. Health sector and primary health care (PHC) services were guided by Zambia’s National Health Policy (2012), sixth National Development Plan (2011–15), and the National Health Strategic Plan (NHSP) (2011–15). The Child Health Policy and the Comprehensive Multi-Year Plan (cMYP) outlined specific strategy, vision and policy for immunization. The cMYP guided strategies and activities for immunization implementation as well as resource mobilization and partnerships based on the NHSP, but was not linked directly to action plans or programme management. Figure 1 illustrates the organizational structure of EPI and the EPI functions of each level.

*General health sector planning and budgeting* was subject to a Medium Term Expenditure Framework with 3-year cycles and annual updates. Annual operational planning at the national level was guided by a well-established, bottom-up system of MoH Action Planning Guides specific to district, facility and community levels. Documents and informants revealed that *vaccine procurement and supply plans* were developed at the national level, informed primarily by collating quantities identified in routine plans. Required vaccine volumes set by the MoH (typically based on international standards) were specified in district-level planning tools. However, related costs and budgets were not. *Human resources planning and budgeting* were primarily determined by central-level processes. However, district-level managers had more direct authority over the compensation of community health workers, districts and facilities.

*Facilities and infrastructure planning* was also controlled centrally. Requests for new facilities were submitted from districts and judged against standard population and distance norms for placement of each type of hospital, health centre and health post. Districts and facilities submitted procurement requests for vehicles—a key determinant of immunization costs and coverage—to higher levels, which then prioritized requests to be granted when budgets became available. Most often, vehicles were funded by donors. *Performance assessment* at district and facility levels was guided by an established performance management system reporting several immunization indicators: achievement of 80% full immunization by 1 year of age, availability of protocols and certain cold chain inputs, and implementation of processes, such as record checking and community-based strategies.

**Box 1: Eight key planning and management systems identified**

- MoH Action Planning Guides
- Financial planning and management systems
- EPI reporting system
- Performance review system
- Vaccine supply and stock management systems
- Human resources information systems
- Facilities and infrastructure planning systems
- Capital equipment planning and procurement procedures

### Using costing information to enhance EPI performance: opportunities and constraints

#### Systems

The MoH Action Planning Guides for the district, facility and community levels were noted to be central to the bottom-up planning system for the health sector in Zambia. ‘Health is the only sector that has a bottom-up planning system’, one MCDMC planner reported. These complex tools encouraged managers to consider processes and budget requirements to achieve targets, which presented opportunities to improve the efficient use of resources. However, the guides were not specifically focused on immunization, or on improving efficiency. The guides focused on the availability of inputs, and on outputs and coverage: there were no indicators of operational or allocative efficiency to link these inputs with outputs or coverage, such as doses administered per staff member or per outreach session. Existing immunization-specific guidance outlined budget allowances for staff and CHWs, transport and maintenance, but lacked direction for large immunization-related costs, such as vaccines. Overall, the guides gave no explicit direction on how to use available resources and strategies most efficiently.

The Financial and Administrative Management System (FAMS), developed by the Central Board of Health in the 1990s, allowed budgeting and tracking of expenditures across all PHC services, including immunization. However, respondents reported that this spreadsheet-based system has had limited capacity to interface with the broader public financial management system working across sectors beyond health. They highlighted that the system lacked basic features, such as budgeting templates, and did not include budgeting for key resources, such as staff and vaccines at the district level. Respondents stated that these constraints were compounded by the inexperience of many accounts staff. Consequently, managers had limited ability to identify actual expenditures on immunization and other activities or the relative value of vaccines, staff and other resources allocated to them. For example, participants suggested that routine comparisons between financial or other resource use and EPI programmatic output or coverage data could improve assessment of EPI budgets and expenditure for the Child Health programme.

*It would be useful to have such cost information for facilities more often to increase awareness of resource use and management* (PMO).

*[Access to cost data] helps to be able to benchmark against other facilities and districts […] for performance and budgeting that weighs rural service needs [appropriately], for example, for fuel and transport* (DMO).

The EPI reporting system provided key statistics for assessing efficiency and performance for immunization and even other services. A PMO noted that, ‘Immunisation data is a very useful indicator of performance. We use EPI data and quarterly trends to guide targeting of supervisory visits’. However, a number of national, provincial and district respondents were concerned about the completeness and accuracy of EPI reporting and analysis as well as the limited use of EPI data at the service level.

Managers generally reported that the established performance review system and tools effectively identified problems and remedial actions. Still, it did not attempt to assess *efficiency of resource use*, focusing on inputs and outputs separately rather than in efficiency or unit cost indicators. One PMO reported the ability to reallocate resources to prioritize efficient use, but a DMO argued that decisions from higher levels did not accurately reflect local priorities: ‘Central decisions often give you unnecessary things, not priorities like vehicles or motorbikes. Often, a poor decision on equipment brings extra running cost, not just the equipment cost’. Budget constraints had also made it increasingly difficult to feed results back to districts to help assess and remedy performance problems.

The centralized vaccine procurement and budgeting system was noted to constrain current efficiency, but offer potential to improve performance in managing these high cost and coverage-determining resources. However, the focus was on quantities rather than cost in planning tools. As a result, managers have had limited knowledge of the relative costs of vaccines, which may adversely influence incentives for efficient use and subsequent management decisions. Furthermore, limited wastage information and poor stock records made it difficult to assess increased the risk of stock outs and vaccine wastage—a significant concern in five of the sampled districts.

*The most exciting thing about the costing study is that it brings the focus back to staff costs and inefficient use of them. It emphasizes that we must not neglect HR and vaccines just because they are paid centrally* (PMO, supported by two DMOs).

Human resources management systems were reported to both hinder and accommodate efficiency efforts. The information from human resources information systems was reported annually and managers and planners had limited access to up-to-date data to compare staffing and costs to workloads for specific services. Furthermore, respondents reported that data might not have reliably reflected staff movements and the actual current distribution of staff. Overall, this presented significant constraints on ability to assess and optimize the allocation of staff. Nevertheless, other respondents indicated that these data were indeed used at the provincial level for active planning: ‘The provinces do calculate staff to population and staff to workload ratios every 6 months and [use] them to plan and adapt staff allocations’.

The costing study had shown that poor facility placement was a major structural reason for inefficient outlier services. Many respondents indicated that facilities and infrastructure planning systems had been relatively successful in ensuring consistency in service allocation based on population and distance, limiting structural inefficiencies. However, some duplication, inefficient location of facilities such as health centres or posts, or modification of services still occurred. As a respondent outlined: ‘A major inefficiency is decisions to over-invest in facilities by politicians or outsiders. Then, recurrent resources also follow, even when not justified by the population’. Several PMO and DMO teams concurred that such allocation resulted in negative impacts on service unit costs.

Systems for planning and procurement of capital equipment, such as vehicles, were noted by several districts to have limited responsiveness. Requests submitted by districts to higher levels were not met in a timely or reliable way, even when absence of equipment produced serious performance bottlenecks. Respondents also noted that no long-term plans or budgets for reliable replacement of capital equipment over time were in place.

#### Structure

Despite Zambia’s historical promotion of decentralization and bottom-up planning, district and service managers had limited control over key budget items and functions. Managers reported that they could not actively manage items outside of their direct budgetary authority, including human resources and vaccines. A central MoH planner stated, ‘In theory, there are bottom-up decisions in management, but particularly for immunization, big decisions affecting resource use are made centrally’.

Several district managers echoed a DMO who stated, ‘We cannot manage HR and vaccine costs, which are managed centrally, [so] there is not much point in telling us about them’. Importantly, however, several PMOs and DMOs noted that provincial and district managers can reallocate staff within and between services, despite having limited influence over the overall staff establishment, planning and budgeting of staff resources. As discussed above, available data could also generate staff workload ratios, allowing managers to identify and investigate outlier services with over- or under-staffing.

#### Strategy

Zambia has consistently prioritized immunization in health and development strategies. Immunization coverage has been one of three key performance indicators for the health sector overall, and there was emphasis on accountability for EPI at all levels. This environment suggests that services will be supported to optimize EPI information and performance given available resources, and immunization strategies will be modified based on evidence describing ways to enhance EPI performance and efficiency. In most districts, commitment to outreach was considered a ‘non-negotiable’ part of strategy. Participants saw this as a key reason coverage was maintained even as resources declined, thereby increasing efficiency with limited need for better information. Even in urban districts with high coverage, managers were reluctant to consider that the costs of outreach to achieve small extra improvements in coverage might not be an efficient use of resources.

#### Style of leadership

Respondents highlighted that performance of districts and facilities depended heavily on the quality of leadership provided by DMOs and their teams. However, at least three DMO team discussions reported that leadership was highly variable given frequent turnover of strong managers.

#### Staff capacity and skills

Despite expressing concerns about declining and unpredictable funding, experienced managers showed capacity to interpret and possibly generate cost and efficiency indicators. They also showed capacity to act on likely inefficiencies, even in the absence of quality data. For example, in response to staffing limitations, some managers shifted tasks, such as recording statistics to CHWs, while others reported increasing reliance on telephonic supportive supervision. There were also differences between capacities for efficient resource allocation reported by provincial- and district-level respondents. The following statement illustrates managers’ interest, but limited capacity, in incorporating efficiency considerations into decision-making:

*Efficiency assessment is not done as the routine data collection is not there and it is not easy to collect information* (PMO).

Other efforts to improve efficiency included reducing outreach expenses by improving coordination of teams’ trips and sessions, increasing community mobilization and reducing outreach to urban communities that already have high coverage. Less experienced and skilled managers had limited abilities in budgeting, planning and expenditure tracking. Respondents pointed to the high turnover and poor morale among skilled managers and health workers as contributing factors to skill deficits, particularly in districts further away from main centres.

Respondents identified capacity building for planning and management as a strong feature of the Zambian system, involving extensive orientation, mentoring and supportive supervision of lower levels by experienced managers during the annual Action Planning and budgeting process. However, supportive supervision, mentoring and training on planning tools had become less frequent in recent years, due to limited financial resources: ‘We sacrifice supervision visits when funds are short [as] we can do it by phone’, said a DMO. A national-level manager explained that, ‘It is not just because it is not in their budget that stops active management. It is not enough to supervise. You have to teach staff to use planning tools and manage resources and vaccines efficiently’. A PMO explained that weaknesses in management capacity are accounted for by higher levels: ‘Management skills to cope with budget ceilings, etc. are generally okay, but vary, especially at facility level, but new and weak [managers] are always guided and we pick up any mistakes’.

Respondents identified other specific capacity gaps, including overburdened MCH coordinators, inadequate training of accountants in use of FAMS, uncoordinated task shifting to CHWs, limited cold chain management expertise and limited capacity among nurses and/or pharmacists in vaccines supply management.

#### Shared values

Respondents identified commitment of managers and services staff to service performance, efficiency, teamwork and mutual support as well as ability to use scarce resources optimally to be an important facilitator of performance, with a DMO stating, ‘This is a very big issue. Staff concern about wasting doses and turning people away’. Many districts reported that service staff and CHWs continued immunization outreach to maintain service coverage, even when they could not be assured of being paid allowances under current financial constraints.

Mentorship and supportive supervision in planning and implementation was reported to be important for helping managers to improve system performance. However, respondents highlighted that diminished funding, particularly for transport, was putting supportive supervision and mentorship at risk. Per diem and travel allowances often created incentives for staff to undertake outreach and supervisory activities. They were generally considered to help to maintain morale among staff and to contribute to immunization coverage and performance. Respondents were divided over whether these incentives actually increased expenditures and inefficiency, by, e.g., increasing inappropriate use of costlier outreach service models.

#### Other factors

Unpredictability of the timing and completeness of disbursement of budgets to districts and services was a major overall constraint on performance. Key inputs, such as fuel and maintenance, were not reliably available for services and exacerbated the decline in resources for supportive supervision, mentorship and training. Respondents reported that these uncertainties and chronic underfunding also undermined both their ability and motivation for active planning and management, and ability to improve efficiency.

Outreach services were discussed at length with varying opinions on whether or not it may be feasible to act on costing information given limited budgets. At least one PMO and three DMOs reported challenges in managing outreach when understanding costs. As a DMO describes, ‘We analysed allowance costs and doses from outreach and concluded that we can’t do without outreach and can’t change routes and schedules’. Another reported that, ‘Rural people need basic education and to have costs of attendance reduced. Otherwise, coverage suffers’. While a DMO described that, ‘Immunisation is just one of the important things we do in outreach. There is greater efficiency in combining trips and activities to deal with low resources’, at least one PMO and one DMO disagreed, arguing that, they ‘*can* manage outreach performance with fixed resources: combine routes, strengthen community mobilization to increase uptake’. Through these discussions, outreach activities were understood to be critical to maintaining coverage as well as major challenges in decision-making with the aim of achieving cost efficiency.

## Discussion

There were opportunities for local managers to make decisions towards enhancing performance within the existing structure and organization of Zambia's immunization programme (Table 1). Results described managers’ capacity to use costing information to generate cost and efficiency indicators, which were not already provided by existing systems and processes. As discussed in interviews, this, in turn, would allow them to understand the relative value of vaccines and other resources, increase awareness of resource use and management, benchmark against other facilities and districts, and modify strategies and priorities towards improving programme performance and efficiency. These opportunities to enhance planning and management of the immunization programme using costing information were tempered by constraints, including limited capacity of human resources, restricted authority to make relevant decisions in several cases, few linkages between immunization costs and its influence on other sectors, and overall budget constraints.

**Table 1:** Summary of opportunities and constraints of enhancing immunization programme performance

Dimension	Opportunities	Constraints
Systems	Established systems encourage managers to consider processes and budget requirements needed to achieve targets RED planning, budgeting and tracking systems in place for immunization Established EPI statistics reporting system Performance review system identify problems and remedial actions Facilities planning norms encourage consistency in service resource allocation	MoH Action Planning Guides do not focus on immunization Performance review does not assess cost and efficiency issues No linkage of inputs to outputs or coverage indicators No consideration of immunization-related costs No flexibility in budget templates Incompleteness and inaccuracy of EPI reporting system Limited feedback on performance Limited systems for up-to-date staffing and staffing costs information Limited systems to increase knowledge of vaccine costs among local managers Limited responsiveness of systems for capital procurement
Structures	Authority to reallocate staff within and between services Can investigate instances of over- or under-staffing	Centralization of decision-making for immunization Limited control over key budget items and functions (i.e. human resources and vaccines) leads to passive approach by local managers
Strategies	Consistent prioritization of immunization in national health policy Supportive environment to improve efficiency of immunization programming within resource constraints Further research advocated to ensure effectiveness of newer policies	
Style of leadership		Performance highly dependent on the quality of leadership provided by DMOs and their teams High turnover of strong managers
Staff capacity and skills	Managers able to interpret and generate cost and efficiency indicators Managers able to identify ways to reallocate scarce resources to enhance performance Extensive orientation, mentoring and supportive supervision of lower levels and experienced managers	High turnover of skilled managers Poor morale of staff Limited availability of experienced accounts staff Low capacity at sites farther from main centres Declining funding for supportive supervision and mentoring
Shared values	Staff commitment to EPI services Support for supportive supervision and mentoring	Divisions of perspectives on the value and function of staff incentives
Other factors		Unreliable disbursement of budgets

RED: Reaching Every District in Zambia with High Quality Routine Immunization Services.

Managers further highlighted priorities for immunization, such as potential for more efficient use of human resources, vaccines and outreach—issues that are often under-emphasized or seen as beyond their influence. Managers reported having data and information necessary to identify problems and make informed decisions using well-established systems for planning and management. Still, further improvements could be realized in terms of availability, reliability and timeliness of data.

Despite decentralization and delegation of decision-making power, there were considerable limitations on the range of decisions that local-level managers could make to improve immunization programme efficiency: major resource allocation decisions were made centrally and existing planning tools were not constructed with a focus on immunization-related costs, providing little guidance on strategies for efficient resource use for immunization. While the consistent high-level prioritization and commitment to immunization were encouraging, bottom-up planning and decision-making for immunization resource use seemed to be limited.

This study reveals the capacity among local-level actors in Zambia to use costing information and identify inefficiencies with actionable solutions for improvement. Yet, overarching constraints that managers face, such as unreliable budget disbursement, may require higher-level reforms and decisions to achieve a durable solution. Such constraints convincingly demonstrate the interconnections of state and national authorities with the decision space of local agents, consistent with the complexity theory perspective taken here. However, they also highlight the viable potential for central and lower level authorities to relieve constraints on local action to improve service performance.

Changing formal systems or tasks, such as supportive supervision, receiving training by more experienced managers, or adjusting systems could also generate more indicators around efficiency issues. It could also be possible to better mediate relationships that involved disparities in power, interests and potential conflicts between management levels (Boesen and Therkildsen, 2005). Opportunities include negotiating prioritization of immunization when funds for transport or allowances are limited or tackling arbitrary delays and budget cuts in order to improve financial flows for immunization. The potential to change performance mainly using existing capacity and systems has also been raised by recent results-based financing pilots in Zambia, which suggested that simply providing small, flexible supplementary budgets to districts can enable managers to enhance performance of immunization and other priority public health services (Friedman *et al.*, 2016).

Examining Zambia’s immunization activities using the framework of complexity theory allows for a broader consideration of factors affecting performance. Firstly, it shows the limits of conceptualizing the issue simply in terms of centralized and decentralized systems. Secondly, it prompts consideration of issues beyond the traditional paradigm driving costing studies (i.e. a focus on evaluating value for money based on inputs and outputs). Decentralization is typically undertaken on the premise that local agents have richer and more locally specific information upon which to make programme decisions. However, the case of Zambia demonstrates the constraints (and some opportunities) that local managers face beyond limitations related to the quality of data and information that is often the sole focus of costing studies. Study findings also point to the importance of managers’ experience, availability and actual use of appropriately designed planning tools, consistent quality of higher-level leadership and sufficient authority over key budget items and functions.

These factors are key moderators of the utility of costing data to improve programme performance. In many contexts, the best way to leverage the insights provided by costing studies may not be to improve financial management systems and cost-related information *per se*. There may be opportunities to achieve more rapid results by mobilizing use of other existing systems to improve performance and by tracking some more basic indicators such as ratios of staff to utilization or programme outputs. Failure to recognize system constraints may also mean that investment in financial or economic information may yield limited benefits. They also point to the limitations of being too immunization-specific in defining challenges and opportunities. Seeing performance issues in the context of systems and capacity for managing and planning more comprehensive PHC services may be more fruitful.

These conclusions are consistent with the findings of other studies focused on local decision-making for immunization-related activities. A study evaluating efforts to strengthen district-level vaccine-preventable disease surveillance and response activities after decentralization in Georgia highlighted lack of authority among local officials, limitations on government resources to carry out activities and unresponsiveness of higher levels as potential barriers to enhanced performance (Hotchkiss *et al.*, 2006). Experience in Cambodia has illustrated how reforms of immunization planning systems, focused on strengthening national programme decision-making and broader stakeholder participation, were among the interventions associated with significant improvement in public health programme performance (Soeung *et al.*, 2007). A previous review of decentralization of health systems more generally not only reported improvements in planning processes, but also increased coordination challenges. Data from the review consistently showed negative effects of decentralization on the availability of vaccines or other medical equipment as well as poor performance attributed to low technical and management capacity (Cobos Muñoz *et al.*, 2017). Recent decentralization of the health system in Kenya has highlighted opportunity for local-level prioritization, but subsequent research on its impacts strongly emphasizes the importance of establishing appropriate structures and capacity at local levels to achieve desired outcomes (Barasa *et al.*, 2017; Tsofa *et al.*, 2017; McCollum *et al.*, 2018).

The EPIC Study demonstrated the substantial impact of costing study data on national-level planners’ and managers’ awareness and ability to identify priority costs and efficiency issues. Potential to inform district and facility level management was also demonstrated. However, this study suggests that steps to enhance immunization programme performance via local decision-making should expand beyond costing and reporting activities to include processes of participatory engagement and active capacity building among local managers. For example, the sharing of ideas and discussion that the dissemination workshop facilitated helped managers to consider active management of key resources in new ways, confirm conclusions, and understand implications. Ongoing participatory engagement of managers is desirable to translate information, develop practical management responses and fine tune capacity building tools and exercises to local needs. Learning from peers was a key advantage of the multi-district workshop format for feedback, echoing the findings of other studies on enhancing evidence-informed policymaking (Langlois *et al.*, 2016). Structured processes for peer interaction, such as regularly planned workshops, promote the exchange of learning-by-doing experiences and of tacit knowledge across settings and programmatic areas.

Potential limitations of this study include the restricted sample size in terms of districts, structure of the study dissemination workshop that combined district- and provincial-level management teams over a 1-day period, and lack of anonymity or confidentiality in group interview settings that may affect responses. However, respondents were generally forthcoming in expressing views, and the responses and priorities that they emphasized were generally consistent between them. There was also limited opportunity to explore which bottlenecks to performance or opportunities should be prioritized and how they could best be operationalized, as that would have involved a substantive process in itself. Finally, this study focused solely on public provision of immunization services, though private providers also deliver and finance these services.

Further research should explore which approaches involving local decision-making for performance improvement yield the largest, quickest benefits. One priority is to understand the extent to which immunization-specific costings and interventions can enhance immunization (and potentially other PHC services) performance at local level, and where more comprehensive PHC management and planning approaches will produce better health system results. Local-level management may prove beneficial in the long term, but face challenges in the short term due to factors such as management turnover. Informants suggested that existing systems for supportive supervision, performance management to target priority deficits, and peer learning or ‘twinning’ with more experienced managers may be important to limit short- and long-term effects of turnover. Evaluations of which models of such interventions are most resilient, practical and cost effective in resource-constrained contexts would provide a useful basis for prioritizing support and clarifying the complementary role of financial management changes.

Routine generation and use of an ambitious, detailed set of cost and output data and indicators may be a difficult way of managing efficiency in the reality of many Zambian districts. Simpler approaches may be more feasible and useful in the short-term. These include improving reliability and use of routine EPI and staffing indicators, better vaccine stock management, and drawing on general insights from periodic costing studies. Additionally, it is worth noting that focusing on costs may be interpreted as a focus on cost-cutting, which can be more challenging to the morale and technical capabilities of health managers and staff. Thus, concentrating efforts on maximizing immunization coverage with available resources may be desirable.

## Acknowledgements

We are grateful to the Provincial and District management teams who participated in the process of disseminating costing study results and exploring their ability to use them, as well as for the guidance and information provided by members of the Planning and Maternal and Child Health functions in the Ministry of Health, Zambia.

## Funding

The original costing study (EPIC1), dissemination of its findings to stakeholders (EPIC2) as well as this study and its open access publication (EPIC3) were made possible by funding from the Bill & Melinda Gates Foundation.

*Conflict of interest statement.* None declared.

## Supplementary Material

czz039_Supplementary_Appendix
