# Supplementary material for: Incorporating costing study results into district and service planning to enhance immunization programme performance: a Zambian case study
Source: Health Policy Plan. 2019 Jun 3;34(5):327–36. doi: 10.1093/heapol/czz039 (PMC6736183; doi:10.1093/heapol/czz039)
Supplement: czz039_Supplementary_Appendix [file czz039_supplementary_appendix.docx]

**Appendix: Policy Documents and Guidelines Reviewed**

1. The Central Board of Health (1996). Financial and Administrative Management System (FAMS).
2. Ministry of Community Development, Mother and Child Health. Strategic Plan for the Ministry of Community Development, Mother and Child Health 2013-2016. Republic of Zambia, Lusaka.
3. Ministry of Finance (undated). Financial Management System (IFMIS) chart of accounts.
4. Ministry of Finance (May 2014) National Planning and Budgeting Policy: Responsive, Transparent, Accountable and Results-Oriented Development Planning and Budgeting Processes. Republic of Zambia, Lusaka.
5. Ministry of Finance (2014). Revised Sixth National Development Plan (2013-2016). Republic of Zambia, Lusaka.
6. Ministry of Health (2009). Action Planning Handbook for District Health Teams (5th Edition April 2009). Republic of Zambia, Lusaka.
7. Ministry of Health (2011). Action Planning Handbook for Health Centres, Health Posts and Communities (6th Edition). May 2011. Republic of Zambia, Lusaka
8. Ministry of Health (2011). Action Planning Handbook for MoH Headquarters and Provincial Offices (2^nd^ Edition May 2011).
9. Ministry of Health, Zambia. (2011). Effective Vaccine Management: Towards improving the Immunization Supply Chain Management in Zambia. July 2011. Republic of Zambia, Lusaka.
10. Ministry of Health (2011). Operational Implementation Manual for Results Based Financing (RBF) in Pilot Districts in Zambia. Republic of Zambia, Lusaka
11. Ministry of Health (2011). Zambia Comprehensive Multi Year Plan (2011-2015): Immunization Vision & Strategy. Republic of Zambia, Lusaka.
12. Ministry of Health (2011). Zambia National Health Strategic Plan (2011-2015). Republic of Zambia, Lusaka.
13. Ministry of Health, Zambia. (2011). Zambia Vaccine Cold Chain Scale-up Strategy. Republic of Zambia, Lusaka.
14. Ministry of Health (2012). Zambia’s National Health Policy. Republic of Zambia, Lusaka.
15. Ministry of Health, Zambia. (2013). Annual Health Statistics Bulletin 2011. Republic of Zambia, Lusaka.
16. Ministry of Health & CHAI (2015). Minutes and Presentation: Strengthening District Systems for Monitoring Financial and Programmatic Performance: Integrated Budgeting and Accounting Tool. FAMS Enhancement Workshop 1st – 2nd March 2016, Gonde Lodge Kabwe.
17. Ministry of Health (2016). Integrated Budgeting and Accounting Tool (Excel Workbook: 10.03.2016).
18. Ministry of Health (Undated – as used in 2015). Performance Assessment Tools for PMO, Districts, Health Centres and Hospitals (Excel Workbook). Republic of Zambia, Lusaka.
19. Ministry of Health (Undated) Reaching Every District (RED) in Zambia with High Quality Routine Immunization Services: Revised Health Facility Action Plan Tool. (Include planning and budgeting tools).
20. Ministry of Health (Undated). Routine EPI programmatic reporting tools for facility and district levels.
21. Ministry of Health (undated). Vaccine supply and stock control tools. (Generally based on standard WHO supply and stock management tools and approaches).
22. WHO (2008). Implementing the Reaching Every District Approach: A Guide for District Health Management Teams. Revised August 2008. WHO Regional Office for Africa.
23. WHO (2014). Comprehensive Multi-Year Planning (cMYP): A Tool and User Guide for cMYP Costing and Financing. Update 2014. Department of Immunization, Vaccines and Biologicals. Geneva.
24. World Health Organization. Logistics Planning Tool spreadsheets (<http://www.who.int/immunization_delivery/systems_policy/logistics/en/index4.html>).
25. World Health Organization. Vaccine Volume Calculator spreadsheet (<http://www.who.int/immunization_delivery/systems_policy/logistics/en/index4.html>).
26. World Health Organization. (undated). Zambia's immunization costing and financing situation. Retrieved online on 4th August 2013 from <http://www.who.int/immunization_financing/countries/zmb/about/en/>.
